# MYB regulator of “colorless” flavonols underlies the evolution of red flowers in *Iochroma* (Solanaceae)

**DOI:** 10.1093/g3journal/jkaf230

**Published:** 2025-09-30

**Authors:** Lucas C Wheeler, Maximilian Larter, Stacey D Smith

**Affiliations:** Department of Ecology and Evolutionary Biology, University of Colorado Boulder, Boulder, CO 80309, United States; Department of Ecology and Evolutionary Biology, University of Colorado Boulder, Boulder, CO 80309, United States; National Research Institute for Agriculture, Food and Environment (INRAE), BIOGECO, Université Bordeaux, Pessac 33615, France; Department of Ecology and Evolutionary Biology, University of Colorado Boulder, Boulder, CO 80309, United States

**Keywords:** transcriptomics, flavonoid biosynthesis, pigmentation, flower color, pelargonidin, gene regulation

## Abstract

Anthocyanins, the pigments that give rise to blue, purple, red, and pink colors in many flowers and fruits, are produced by the deeply conserved flavonoid biosynthesis pathway. The regulation of this pathway is thus fundamental for species differences in color across flowering plants, and a growing body of evidence implicates MYB transcription factors as key players activating or suppressing the production of different pigments. Nevertheless, the potential role of MYBs genes in determining the type of pigment produced (as opposed to the overall amount) is unknown. Here, we demonstrate that a lineage of R2R3 MYBs that is closely related to well-known flavonol regulators (MYB12 members in subgroup 7) is the primary determinant of the shift from blue to red flowers in the genus *Iochroma*. Similar to its ortholog in *Capsicum*, this *Iochroma MYB12-like* gene controls the expression of flavonoid-3′-hydroxylase, the pathway branch point between red and blue pigments, and when down-regulated, results in redirection of flux toward red pigments. These results underscore the importance of transcription factor evolution in generating phenotypic novelty as well as the competitive nature of interactions among flavonoid pathway branches. In addition, our study demonstrates the effectiveness of RNAseq of segregating populations, in combination with other lines of evidence, for identifying novel functional variation.

## Introduction

Phenotypic differences between species are often controlled by differences in the timing and patterns of gene expression (Kimura et al. 2008; Des Marais and Rausher 2010; Byers et al. 2014). These differences in gene expression can arise through a variety of mechanisms, including changes in the *cis*-regulatory regions controlling expression (i.e. promoters, enhancers), changes in the expression or function of transcription factors, or post-transcriptional regulation (e.g. gene silencing). Many authors have argued that the *cis-*regulatory mutations will be favored during evolutionary transitions due to their modular architecture, allowing for altered expression in one context without pleiotropic effects in other contexts (Prud'homme et al. 2006; Wray 2007). However, functional changes in transcription factors can have similarly narrow consequences, depending on their specificity in terms of target genes and spatiotemporal patterns of expression (Lynch and Wagner 2008; Panchy et al. 2016; Auge et al. 2019).

Plant MYB transcription factors comprise a prime example of a large and diverse gene family with highly specialized functions. Whereas animal and fungal genomes house at most a few dozen MYB genes, plant genomes contain hundreds of MYBs, even in diploid species (Shiu et al. 2005; Feller et al. 2011; Gates et al. 2016). This expansion of MYB copies in plants is coupled with a diversification of functional roles, from defense, to coloration, to morphology (Ramsay and Glover 2005; Wu et al. 2022). Closely related MYBs often share similar regulatory functions, e.g. as activators or repressors of particular sets of target genes, but vary in their expression patterns, resulting in similar phenotypic effects albeit in different tissues or developmental stages (e.g. Millar and Gubler 2005; Stracke et al. 2007). Nevertheless, with the multitude of MYBs in every plant genome, new functional roles and patterns of diversification are continuing to be discovered (Sagawa et al. 2016; Gates et al. 2018; Mu et al. 2024).

**Fig. 1.**
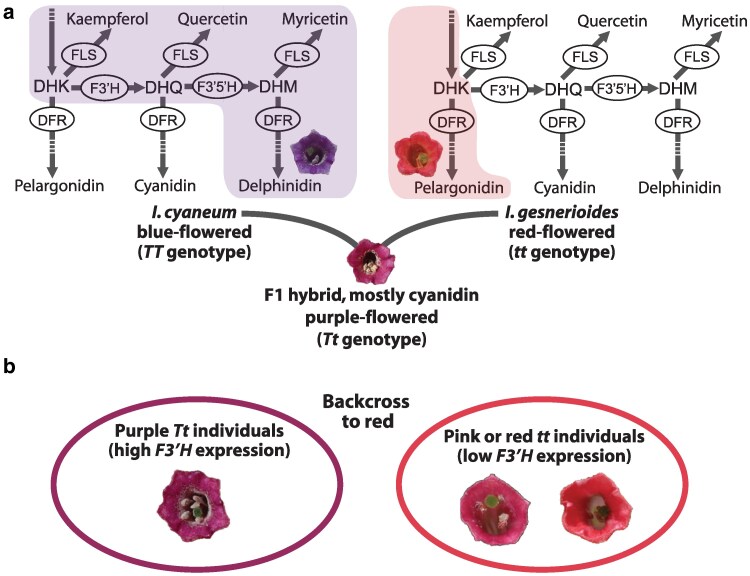
Flavonoid pigment production in parental lines and experimental design for identifying the *T*-locus. a) Segregating backcross populations were created from parental lines of the blue-flowered *Iochroma cyaneum* and the red-flowered *I. gesnerioides*. The former makes delphinidin-derived anthocyanins and all 3 of flavonols (kaempferol, quercetin, and myricetin) while the latter makes only pelargonidin-derived anthocyanins and trace amounts of kaempferol. The active branches of the pathway are shaded in each case. The enzymes shown (in ellipses) are flavonoid 3′hydroxylase (F′3H), flavonoid-3′5′-hydroxylase (F3′5′H), dihydroflavonol reductase (DFR), and flavonol synthase (FLS). Flavonoid intermediates are dihydrokaempferol (DHK), dihydroquercetin (DHQ), and dihydromyricetin (DHM). Additional steps upstream of DHK (e.g. involving chalcone synthase [CHS], chalcone isomerase [CHI], and flavanone hydroxylase [F3H]) and downstream of DFR (e.g. involving anthocyanidin synthase [ANS], glucosyltransferase) are not shown but indicated with the dashed portion of the arrows. Note that F3′5′H has 3′ activity and can act on DHK in some taxa, but in *Iochroma*, it is specialized for DHQ (Smith and Rausher 2011). The F1 hybrid produces mainly cyanidin-derived anthocyanins and is presumed to be heterozygous at the *T*-locus, which controls *F3′H* expression. b) Phenotypes and pools for RNASeq experiment. We divided the backcross population (F1 crossed to the red parent) into a high *F3′H* expression purple-flowered cyanidin-producing pool (presumably *Tt*) and a low *F3′H* expression mostly or entire pelargonidin-producing pink to red-flowered pool (presumably *tt*). See Table 1 for more information on sequenced individuals.

Among the subgroups of plant MYB transcription factors, those regulating floral coloration through the production of flavonoid pigments are among the best studied. The MYB activators of flavonoid synthesis fall into several subgroups of R2R3 MYBs, including the subgroup 7 (SG7) genes that regulate the “early” genes of the pathway (e.g. *CHS*, *F3H*) and the branches leading to flavonol production (*FLS*), the subgroup 6 (SG6) genes that regulate the “late” steps of the pathway (e.g. *DFR*, *ANS*) leading to anthocyanin pigments, and the subgroup 5 (SG5) genes that control proanthocyanidin production (Gonzalez et al. 2009; Dubos et al. 2010; Albert et al. 2014;  Fig. 1). Anthocyanins give rise to the red, purple, and blue floral hues, while flavonols can modify these colors as co-pigments and provide UV-absorbing patterns, such as nectar guides and bullseyes (Sheehan et al. 2016; Todesco et al. 2022). Thus, both types of compounds (anthocyanins and flavonols) are important contributors to floral coloration and are often jointly produced in developing petals.

While this general regulatory architecture is well-conserved across flowering plants (Mol et al. 1998; Schwinn et al. 2014), the factors determining the type of flavonol or anthocyanin produced appear more variable across species, and perhaps for that reason, are not as well understood. Both flavonols and anthocyanins are produced at 3 hydroxylation levels (mono-, di-, and tri-) that have different spectral properties, and their relative production depends on the expression of the so-called branching enzymes, F3′H and F3′5′H (Fig. 1). For example, when both enzymes are highly expressed, flowers will produce the tri-hydroxylated flavonoids, such as the blue delphinidin pigments, whereas when these enzymes are not present, flowers will produce the red pelargonidin pigments (Wessinger and Rausher 2012; Fig. 1). The F3′H enzyme, which is responsible for conversion of DHK (the precursor of the flavonol kaempferol and the red pigment pelargonidin) into DHQ (the precursor of the flavonol quercetin and the purple pigment cyanidin), appears to be regulated by subgroup 7 MYBs in *Capsicum* (Wu et al. 2023) and subgroup 6 MYBs in petunia and *Antirrhinum* (Schwinn et al. 2006; Albert et al. 2011). The other branching enzyme, F3′5′H, has been lost in many flowering plant lineages (e.g. morning glories, roses, penstemons; Rausher 2006; Wessinger and Rausher 2012), but in those species which have an intact copy and have been well-studied in terms of anthocyanin regulation, its expression is typically co-regulated with the anthocyanin-specific steps of the pathway by the subgroup 6 MYBs (Albert et al. 2011).

Substrate preference of the multifunctional enzymes also plays a key role in the type of flavonoids produced. While several of the pathway enzymes are able to accept multiple precursors, they often exhibit higher activity for one or a subset of substrates. For example, the petunia DFR enzyme is able to act on all 3 dihydroflavonol precursors (DHK, DHQ, and DHM), but preferentially acts on the latter 2 to produce cyanidin and delphinidin-derived anthocyanins (Johnson et al. 1999). The FLS enzyme, which competes with DFR for dihydroflavonols, also acts a substrate specialist in most taxa with its preference often overlapping that of DFR (Choudhary and Pucker 2024). Nevertheless, the available substrates for both enzymes depend on the activity of F3′H and F3′5′H, which are required for DHQ and DHM production. Moreover, the overlap in function between F3′H and F3′5′H depends on the species; in some taxa, such as the tea plant and some Asteraceae, F3′5′H can carry out both 3′ and 5′ hydroxylation (Seitz et al. 2007; Wang et al. 2014), while in others, such as petunia and *Iochroma*, this enzyme primarily acts at the 5′ position to make DHM from DHQ (de Vetten et al. 1999 ; Smith and Rausher 2011 ; Fig. 1). Thus, the flux through the pathway and the resulting pigmentation depends on the complex interplay of patterns of expression and functionality of these enzymes (Wheeler et al. 2021, 2023).

In the present study, we investigate the regulatory control of *F*3′*H* expression in *Iochroma* (nightshade family, Solanaceae), one of several genera in which red pelargonidin-producing flowers have evolved from blue delphinidin-producing ancestors. Previous work demonstrated that this flower color transition in the red-flowered lineage involved 3 genetic changes, including the floral down-regulation of *F*3′*H*, the loss of the *F*3′*5′H* gene, and a shift to specialization of DFR on DHK (Smith and Rausher 2011; Smith et al. 2013). Among these changes, the loss of *F*3′*H* expression has the largest effect on floral pigmentation because it largely eliminates flux away from DHK, allowing anthocyanin production to be entirely redirected toward pelargonidin (Smith and Rausher 2011; Fig. 1, note again that F3′5′H is specialized for DHQ in Solanaceae). Moreover, this shift in *F3′H* expression is due to a *trans-*regulatory mutation, as the genotype at the *F3′H* locus itself does not predict flower color in segregating populations (Smith and Rausher 2011). This unknown regulator of *F3′H*, which segregates as a single gene, was termed the “*T*-locus” (Smith and Rausher 2011).

Here, we use a suite of genomic, transcriptomic, and biochemical approaches to identify candidates for the *T*-locus responsible for the shift toward pelargonidin production and in turn, the evolution of red flowers in *Iochroma*. Using biochemical and expression data, we first sorted individuals from a backcross population by pigment phenotype and corresponding difference in *F3′H* expression. Next, we searched the floral transcriptomes of these 2 pools of individuals for genes that match the predicted allelic pattern (e.g. homozygous for the red-flowered parent allele in the pink/red-flowered pool) and show the predicted association with *F3′H* expression. Our analyses point to a single R2R3 MYB transcription factor that is related to the MYB12 members of Solanaceae subgroup 7 MYBs but falls in a deeply diverged clade, only functionally characterized in chili peppers. As we discuss, these results suggest that the subgroup 7 MYBs may be much more diverse than previously known and play an underappreciated role in flower color evolution through their effects on flavonol production.

## Materials and methods

### Source populations and phenotyping

Individuals of the blue-flowered *I. cyaneum* were crossed with the red-flowered *I. gesnerioides* to create segregating populations to dissect the genetic basis of their flower color differences (Smith and Rausher 2011). The blue-flowered state is ancestral in *Iochroma* and corresponds to the production of delphinidin-derived anthocyanins, while the red-flowered derived state involves the production of pelargonidin-derived anthocyanins (Fig. 1; Smith and Rausher 2011). The *I. cyaneum* parent was grown from seed from a cultivated accession from the Missouri Botanical Gardens, originally collected by W. G. D'Arcy, and the *I. gesnerioide*s parent was grown from the Solanaceae Germplasm collection in the Botanical Garden of Nijmegen (accession number 944750129). Herbarium vouchers for these accessions are Smith 265 and 266 (WIS), respectively. A single F1 was backcrossed to the *I. gesnerioides* parent, and progeny from the resulting backcross population were genotyped at *F3′5′H* and *Dfr* (Smith and Rausher 2011; Table 1). Anthocyanin production was previously characterized using HPLC and revealed 3 pigment phenotypes (purple-flowered individuals producing primarily cyanidin, pink-flowered individuals producing mostly pelargonidin, and red-flowered individuals producing almost entirely pelargonidin) (Smith and Rausher 2011). The purple-flowered individuals share high *F3′H* expression and are inferred to carry a dominant “blue” allele at a segregating *trans*-acting factor (the “*T*-locus”, Smith and Rausher 2011; Table 1).

**Table 1.** Phenotypes and genotypes of sampled individuals from the backcross population.

Individual	DEL	CYAN	PEL	*F3′H* expression	Inferred *T*-locus genotype	*F3′5′H*	*Dfr*
GCG22	0.2	*0.68*	0.1	High	***Tt***	***F-***	*dd*
GCG55	0.3	*0.63*	0.1	High	***Tt***	***F-***	*dd*
GCG11	0.24	*0.61*	0.15	High	***Tt***	***F-***	*dd*
GCG98	0.2	*0.7*	0.1	High	***Tt***	*–*	***Dd***
GCG84	0.03	*0.67*	0.30	High	***Tt***	*–*	***Dd***
GCG25	0.2	*0.62*	0.2	High	***Tt***	*–*	***Dd***
GCG49	0.12	*0.53*	0.35	High	***Tt***	***F-***	***Dd***
GCG40	0.11	*0.67*	0.22	High	***Tt***	***F-***	***Dd***
GCG94	0.3	*0.57*	0.2	(This study)	***Tt***	***F-***	***Dd***
GCG60	0.01	*0.70*	0.29	High	***Tt***	*–*	*dd*
GCG18	0.01	*0.84*	0.14	High	***Tt***	*–*	*dd*
GCG76	0.05	*0.81*	0.14	(This study)	***Tt***	*–*	*dd*
GCG2	0.24	0.07	*0.69*	Low	*tt*	***F-***	***Dd***
GCG61	0.2	0.14	*0.7*	Low	*tt*	***F-***	***Dd***
GCG23	0.21	0.11	*0.67*	Low	*tt*	***F-***	***Dd***
GCG24	0.2	0.14	*0.7*	Low	*tt*	***F-***	*dd*
GCG73	0.2	0.12	*0.7*	Low	*tt*	***F-***	*dd*
GCG7	0.17	0.14	*0.69*	Low	*tt*	***F-***	*dd*
GCG4	0.05	0.05	*0.90*	Low	*tt*	*–*	***Dd***
GCG85	0.02	0.04	*0.94*	(This study)	*tt*	*–*	***Dd***
GCG6	0.08	0.11	*0.81*	Low	*tt*	*–*	***Dd***
GCG9	0.05	0.04	*0.91*	Low	*tt*	*–*	*dd*
GCG104	0.1	0.08	*0.9*	Low	*tt*	*–*	*dd*
GCG43	0.07	0.06	*0.87*	Low	*tt*	*–*	*dd*

The DEL, CYAN, and PEL columns show the proportion of anthocyanins derived from blue delphinidin, purple cyanidin, and red pelargonidin pigments, respectively (data from Smith and Rausher 2011); the value for the predominant pigment is italicized. The expression of *F3′H* was quantified with qPCR in Smith and Rausher (2011); individuals with “low” expression have 10-fold lower expression than those with “high”. Individuals with high *F3′H* expression and primarily cyanidin production are predicted to be heterozygous at the *T-*locus with one “blue” and one “red” allele (Tt). Three individuals were not included in the previous qPCR experiment; their inferred *T*-locus genotype was based on pigment production (CYAN vs PEL) and their *F3′H* expression was measured as part of this study. Thus, the samples are split between *Tt* and *tt* individuals at the *T-*locus, and there are 3 replicates for each combination of genotypes at the other involved loci (*F3′5′H* and *Dfr*). Note that the red parental species is missing the functional copy of *F3′5′H,* so the red allele is indicated with a –.

### Biochemical phenotyping and RNA-Seq of backcross individuals

We performed RNA-Seq on corolla tissue from 24 backcross individuals segregating for the putative *T*-locus. We sampled 12 individuals with each inferred *T*-locus genotype: *Tt* corresponding to one dominant “blue” allele and high *F3′H* expression or *tt* corresponding to 2 recessive red alleles and low *F3′H* expression (Table 1). We divided these 12 among the possible genotypes at the other 2 loci that affect flower color in this cross (*Dfr* and *F3′5′H*). DFR shows functional specialization, with the red allele specialized for activity on DHK (Smith et al. 2013 ), while *F3′5′H* is absent from the red parent genome (Smith and Rausher 2011). With 4 possible combinations at these other 2 loci (*Dd*/*F*-, *Dd*/−, *dd*/F-, *dd*/−), we sampled 3 biological replicates of each within the groups of 12 (Table 1). We included all possible genotypic combinations at the 3 loci influencing flower color in order to isolate the *T*-locus while balancing across the effects of these other loci. For RNA extraction, we flash-freeze corolla tissue from buds of roughly 1.25 cm in length, which is equivalent to petunia bud stage 5 (Pollak et al. 1993). This developmental stage shows expression of both early and late pathway genes in the anthocyanin pathway (Larter et al. 2018). Total RNA was extracted with the Spectrum Total RNA extraction kit (Sigma, St Louis, MO). Library preparation and 150-base-pair paired-end mRNA sequencing was carried out by Novogene (Sacramento, CA).

### Identifying SNPs associated with flower color and *F3′H* expression

We used the reference genome assembly for *Iochroma cyaneum* (Powell et al. 2022) stored on SolGenomics (Fernandez-Pozo et al. 2015) to call SNP variants and filter the RNASeq dataset for candidate genes for the *T*-locus. RNAseq reads were aligned with STAR v. 2.75b (Dobin et al. 2013), and the resulting BAM files were used as input for the mpileup tool in bcftools v. 1.17 (Danecek et al. 2021). We used the tools mpileup and call in bcftools with default settings to call allelic variants in expressed genes. We filtered variants by base call quality, only retaining variants with quality score greater than or equal to 20. We used the resulting VCF file for subsequent analyses of associations with the color phenotype.

We first split the filtered VCF files into 2 subsets, one for all samples with purple cyanidin-producing flowers (inferred *Tt* genotype at *T-*locus) and one for those with pink or red mostly pelargonidin-producing flowers (inferred *tt* genotype at *T*-locus) (Table 1, Fig. 1a). In order to identify SNPs that differ between these 2 pools, we used *pyvcf* (Casbon 2012) to filter the variants to include only those that are present in all “*Tt*” individuals and not present in any “*tt*” individuals. This strict criterion resulted in a set of SNPs that perfectly co-segregate with the high or low *F3′H* expression (see Results). Most of the SNPs are located on chromosome 5, but some mapped to smaller scaffolds that were not incorporated into the reference assembly (Supplementary Fig. 1). We then used promer from Mummer4 (Marçais et al. 2018) and D-genies (Cabanettes and Klopp 2018) to align these scaffolds back to the *I. cyaneum* and tomato reference genomes.

In addition to this filtering approach, we performed a case-control GWAS with the variant calls in GEMMA 0.98.5 (Zhou and Stephens 2012). We set phenotypes to 0 (purple-flowered *Tt* plants) or 1 (pink/red-flowered *tt* plants) and fit a univariate linear mixed model with the full set of variants. We then plotted the location of all analyzed variants on the assembled *I. cyaneum* chromosomes and scanned variants with significant phenotypic associations using *P* < 5 × 10^−8^ as the genome-wide cutoff (Xu et al. 2014).

### Co-expression of candidate genes with *F3′H*

We predicted that if the *T*-locus is a transcriptional regulator, its expression will likely track that of *F3′H* in the segregating backcross. Thus, we used expression data from the 24 transcriptomes to quantify levels of expression and test for correlations between *F3′H* and loci carrying associated SNPs (previous section). We first created a *de novo* transcriptome for the blue-flowered parent (*I. cyaneum*) to ensure that we captured all expressed genes. For this assembly, we used single-end Illumina RNA-seq data from reproductive, seed, and vegetative tissues from *I. cyaneum* from a previous study (Powell et al. 2022) and assembled the transcripts using the pipeline developed in Wheeler et al (2022). Briefly, this pipeline first corrected read errors in the 128,433,717 raw reads using Rcorrector (Song and Florea 2015) and removed unfixable reads using *unfixable_filter.py* (Yang and Smith 2014) with Python v. 2.17.18. We trimmed adaptor sequences from the filtered reads using Trimmomatic v. 0.39 (Bolger et al. 2014) and used the trimmed reads for *de novo* assembly with Trinity v. 2.11.0 (Grabherr et al. 2011). We removed apparent chimeric sequences using *run_chimera_detection.py* (Morales-Briones et al. 2021), with a reference BLAST database consisting of sequences from *Arabidopsis*, *Solanum*, and *Petunia*. We then used Corset v. 1.09 (Davidson and Oshlack 2014) to cluster transcripts and *filter_corset_output.py* (Yang and Smith 2014) to remove redundant transcripts. Finally, we predicted complete CDS from the Corset-filtered transcripts using TransDecoder v. 5.3.0 (Haas et al. 2013).

Next, we quantified gene expression by pseudo-aligning reads from each backcross individual to the predicted CDS in the transcriptome using Salmon (Patro et al. 2017). We calculated estimated read counts and TPM for each transcript. We imported Salmon quant files, partitioned by inferred *T*-locus genotype (*Tt*/*tt*), into DEseq2 with *tximport* (Soneson et al. 2015) and used the *DESeqDataSetFromTximport* function to create a DEseq analysis object, with treatments corresponding to the *T*-locus genotype. We quantified differential expression between these subsets using the *DESeq* function. We filtered the resulting transcripts by the adjusted *P*-value with a significance threshold of 0.05. This adjusted value accounts for multiple tests through the false discovery rate (FDR, Benjamini and Hochberg 1995); our cut-off corresponds to FDR of 5%.

In addition to examining differentially expressed genes between the phenotypic pools, we also computed pairwise correlations between the expression of *F3′H* and all other genes. We calculated Pearson correlation coefficients across the samples (*n* = 24) and, to account for multiple comparisons, we adjusted the *P*-values using the Bonferroni correction.

Finally, as we expect that any regulator of *F3′H* may also regulate other flavonoid pathway genes, we used WGCNA v. 1.72.5 (Langfelder and Horvath 2008) to identify modules of co-expressed genes, focusing on modules that included *F3′H* and, in turn, are correlated with the color phenotype. WGCNA computes pairwise correlation coefficients, which then are converted to an adjacency matrix with the raw values raised to a soft-thresholding power (**β**) to approximate a scale-free network. For our data, we selected a β of 7, which corresponds to an R^2^ value of 0.88 with the scale-free model and a mean connectivity of 20.4 (Supplementary Fig. 2). We initially used blockwise module detection on the full *de novo* transcriptomic dataset of 19,184 genes, and from this first pass, we retained modules with a correlation of 0.2 or greater with the trait of interest (color phenotype/inferred *T*-locus genotype). The filtered dataset contained 4,854 genes, which allowed us to examine smaller modules (we set the minimum size to 20 genes). After hierarchical clustering, we merged modules that were 90% similar and re-calculated correlations between the module eigengenes and the trait. We exported the module containing *F3′H* to Cytoscape format using *exportNetworkToCytoscape* and extracted the topology overlap matrix (TOM) edge weights. We plotted the distribution of weights for edges containing *F3′H* and for all other edges and used *Z*-scores to capture how extreme each co-expression relationship is within the context of the module.

### Phylogenetic analysis of *MYB12-like* genes and other SG7 MYBs

Our combined analyses of SNP association and gene expression strongly implicated an R2R3 MYB, which we refer to as *Iochroma cyaneum MYB12-like* following the nomenclature in *Capsicum* (see Results). As R2R3 MYBs comprise a large group of functionally distinct transcription factors, we carried out phylogenetic analysis to identify the most closely related copies in other model Solanaceae. We used BLAST searches to retrieve the top hits from tomato, potato, groundcherry, chilipepper*, Nicotiana benthamiana*, and *Arabidopsis thaliana* (as an outgroup) and created a protein alignment with MAFFT v. 7 (Katoh and Standley 2013) using default settings. As BLAST results suggested that the most similar sequences belonged to the flavonoid-regulating subgroup 7 (SG7) MYBs (see below), we included the R2 and R3 MYB domains through to the SG7 motif, which is widely conserved from *Arabidopsis* to tomato (Stracke et al. 2001, 2007; Fernandez-Moreno et al. 2016). The downstream positions were trimmed as they were hypervariable and could not be confidently aligned. We estimated a maximum-likelihood phylogeny using this SG7 amino acid alignment with the best-fitting model of amino acid substitutions (Q.plant + G4) and 1,000 bootstrap replicates in IQ-TREE 2.3.6 (Nguyen et al. 2015; Minh et al. 2020).

Based on this broader phylogenetic analysis, we identified a small set of Solanaceae SG7 MYBs most closely related to the candidate locus. We next estimated a tree from full length coding sequences (CDS) from these closely related copies, which are more easily aligned. We included 6 additional Iochrominae sequences assembled by mapping reads from floral bud transcriptome data onto the *Iochroma cyaneum* genome sequence, again using STAR. Each of these 6 species is represented by 2 biological replicates; a consensus of the 2 was used for the phylogeny and the replicates were used to estimate the levels of *MYB12-like* expression in each species using Salmon as above. We estimated the maximum likelihood tree from the CDS alignment with IQ-TREE, using the best-fitting model of nucleotide substitutions (TIM3 + F + G4).

## Results

### Localization of associated SNPs with flower color in the *Iochroma* genome

**Fig. 2.**
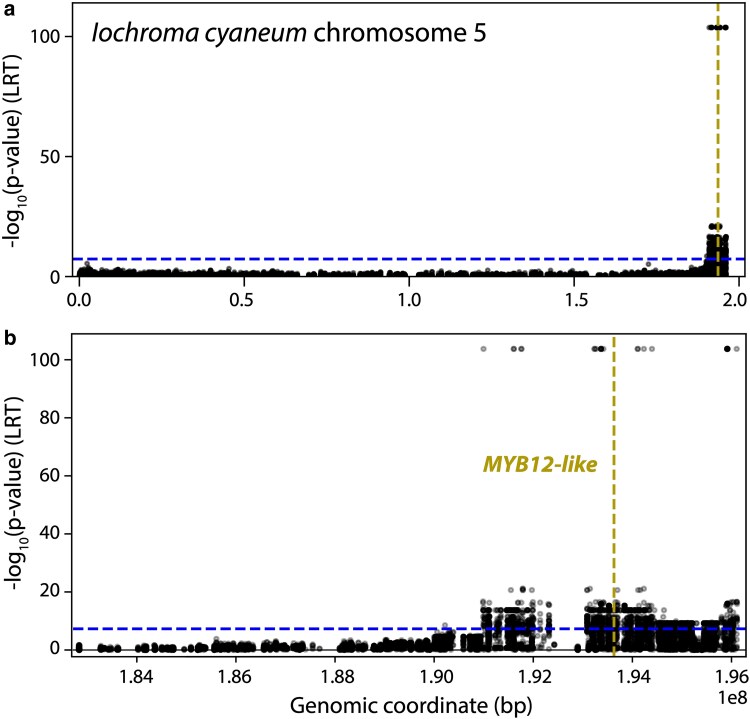
Manhattan plot showing significant SNP associations on *Iochroma cyaneum* chromosome 5. The horizontal blue dashed line marks the cutoff for genome-wide significance (*P* < 5 × 10^−8^). The gold vertical dashed line marks the location of the *MYB12-like* gene, which predicts *F3′H* expression. a) The position of *MYB12-like* near the 3′ prime end of the chromosome. b) Close-up of the region containing *MYB12-like*, showing the concentration of associated SNPS in the last 500 kb of the chromosome.

We recovered 92 SNPs that perfectly co-segregate with the 2 phenotypic pools, i.e. distinguish purple-flowered *Tt* and pink/red-flowered *tt* pools. The majority of these SNPs (49, 53%) fall on chromosome 5 of the *I. cyaneum* reference assembly. We also found 28 SNPs mapping to a roughly 620 Kb scaffold (00085) and the remainder (15) mapping to 3 additional unincorporated scaffolds (Supplementary Fig. 1). Our subsequent analyses suggest that these scaffolds represent segments of chromosome 5 that were not included during the assembly process (Powell et al. 2022). For example, scaffold00085 aligns well with tomato chromosome 5 (Supplementary Fig. 3), and 95% of the CDS retrieved from that scaffold have top hits on tomato chromosome 5. This region appears nested within the larger region of *I. cyaneum* chromosome 5 where most of the SNP associations are clustered (Fig. 2). The 3 smaller scaffolds with associated SNPs (Supplementary Fig. 1) also BLAST to tomato chromosome 5 and were also likely excluded during assembly. Thus, all SNPs recovered from the co-segregation analysis appear to be localized along a small region of *I. cyaneum* chromosome 5.

We carried out a case-control GWAS using the same set of variant calls. This analysis similarly retrieved associations exclusively on chromosome 5, with significant hits in the gene-dense region in the last 500 kb of the chromosome (Fig. 2 and Supplementary Fig. 4). This region of the genome contains 468 gene models (Supplementary Table 1), 352 of which are functionally annotated in the genome (Supplementary Table 2). Twenty-eight of these genes are annotated as transcription factors, and only one corresponds to a known group of flavonoid regulators. BLAST searches suggested this locus (IC05g034110) is a member of the flavonol-regulating subgroup 7 MYBs (Stracke et al. 2007), with top hits to AtMYB111 and AtMYB12 in *A. thaliana* and to MYBs annotated as MYB12 and MYB-like in chilipepper and potato. We will refer to this gene as *Iochroma cyaneum MYB12-like* (*IcMYB12-like*) based on the phylogenetic analysis (see below). The associated region also contains copies of one of the upstream pathway enzymes, chalcone synthase (CHS), as well as UDP-glycosyltransferase (UGT), which can glycosylate various flavonoids.

### Patterns of differential expression and co-expression

**Fig. 3.**
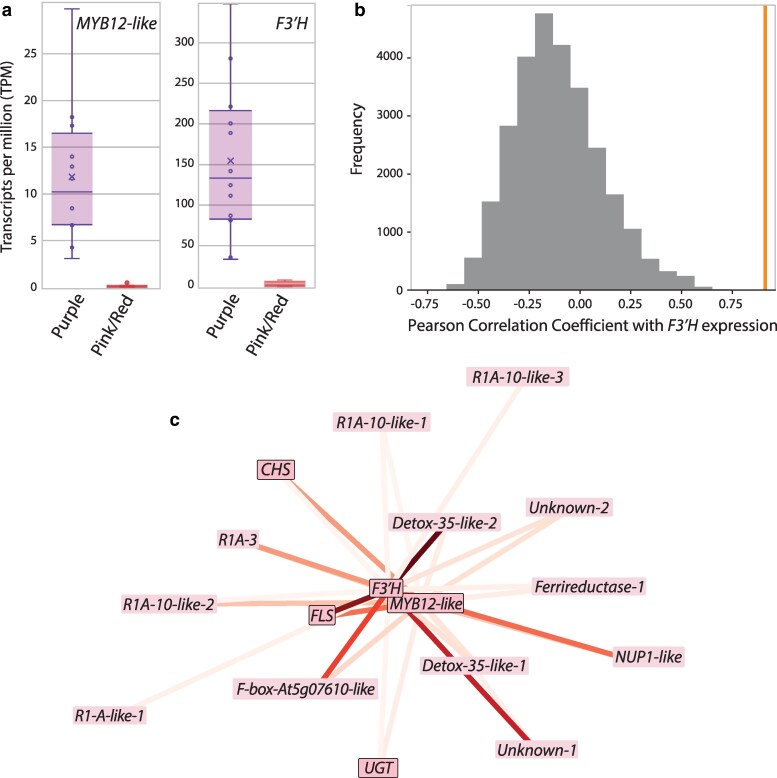
Co-expression of *F3′H* and *IcMYB12-like*. a) Expression levels for each gene in the 2 phenotypic pools. Box plots mark the first and third quantiles, with a bisecting line to indicate the median. The mean is denoted with an x. The Pearson correlation coefficient for the expression of these 2 genes is 0.91187 (*P* = 1.58 × 10^−05^ after Bonferroni correction). b) Histogram of Pearson correlation coefficients between *F3′H* and all other expressed genes. The orange vertical bar marks the value for the correlation between *F3′H* and *MYB12-like*. The value for the correlation with *FLS* also falls at that mark (*r* = 0.91185). See Supplementary Table 4 for complete ranked list of correlation coefficients. c) Submodule from WGCNA analysis containing all edges including *F3′H* and *MYB12-like* (Supplementary Table 5). The lines representing each edge are colored by the connectivity value (TOM) from WGCNA (Supplementary Fig. 6); more closely clustered genes are more tightly co-expressed (i.e. spring layout). Genes encoding enzymes related to the flavonoid pathway (*CHS*, *FLS*, *UGT*) are outlined along with *IcMYB12-like*.

Our DEseq2 analysis identified 58 significantly differentially expressed transcripts between the 2 phenotypic pools in our backcross (Supplementary Table 3). The MYB transcription factor *IcMYB12-like* appears as the sixth most strongly differentially expressed gene between the pools (log_2_-fold change = −6.35, or ca. 82-fold lower expression in the pink/red pool). Its putative target, *F3′H*, is the eighth most differentially expressed (log_2_-fold change = −5.83, or ca. 57-fold lower expression in the pink/red pool) and is tightly correlated to *MYB12-like* (r = 0.91, *P* < 1.6 × 10^−5^, Fig. 3 and Supplementary Table 4). Note that the expression of *F3′H* in many of these individuals was previously measured with qPCR (Table 1; Smith and Rausher 2011); this analysis confirms the strength and degree of the differential expression between individuals presenting the alternate pigment phenotypes (Fig. 3a). A similar degree of differential expression was found for *FLS* between the 2 pools, and 2 other flavonoid pathway genes (*CHS* and *UGT*) also appear among the list of significantly DE genes (Supplementary Table 3). These patterns could indicate some degree of regulatory control of *IcMYB12-like* over other pathway steps.

In addition to examining DE genes between the 2 phenotypic pools, we explored co-expression of genes across the entire set of 24 backcross individuals. If *IcMYB12-like* indeed activates floral *F3′H* expression, we expect the 2 genes to show correlated expression and to belong to the same co-expressed module of genes. Consistent with this prediction, our WGCNA analysis recovered a module of 52 genes containing *F3′H* and *IcMYB12-like* (Supplementary Table 5). Out of the 34 modules found in the analysis, this module is the only one significantly correlated with the pigment phenotype (purple vs pink/red, R^2^=−0.92, *P* = 1e^−10^, Supplementary Fig. 5). Within this module, *IcMYB12-like* is tightly co-expressed with *F3′H* (Fig. 3c). The connectivity between *F3′H* and *IcMYB12-like*, measured as topological overlap matrix (TOM) values from the WGCNA analysis, was the second highest in the set of all edges involving *F3′H* (*Z*-score: 1.96) with only the edge connecting *F3′H* and *FLS* having a higher value (*Z*-score: 2.56) (Supplementary Fig. 6). We also found a tight connection between *IcMYB12-like* and *FLS* (*Z*-score: 1.83), suggesting that both *F3′H* and *FLS* are both regulated by *IcMYB12-like.* Three other flavonoid pathway genes, *CHS*, *CHI,* and *UGT* appear in the module associated with the phenotype (Supplementary Table 5), and all except for *CHI* are directly connected to *IcMYB12-like* (Fig. 3c). Eight other loci within phenotype-associated module are connected to *IcMYB12-like* (*e.g.* DETOX-35-like-2 and the F-box protein At5g07610-like), although no functional connection is known. Three of the genes connected to *IcMYB12-like* (*R1A-10*, the F-box protein *At5g07610-like* and *UGT*) fall in the same genomic region as *IcMYB12-like* (Supplementary Table 2), suggesting that these co-expression patterns may be related to co-localization within the genome (Michalak 2008). Indeed, differentially expressed transcripts are clustered around *IcMYB12-like* (Supplementary Fig. 7). Nevertheless, both *F3′H* and *FLS* occur outside of the region containing *IcMYB12-like* (Fig. 2), excluding co-localization as an explanation for their strong co-expression with *IcMYB12-like*.

### Phylogenetic relationship of *IcMYB12-like* to other MYB transcription factors

**Fig. 4.**
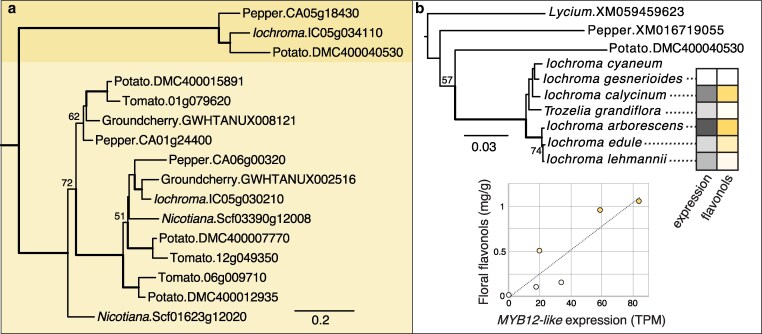
Phylogenetic relationships among Solanaceae subgroup 7 (SG7) MYBs. a) Maximum likelihood phylogeny from amino acid sequences; the sequences highlighted in light yellow include the best studied SG7 MYBs in Solanaceae, e.g., *Sl*MYB12 (Tomato.01g079620) that is tied to the colorless fruit epidermis phenotype (Ballester et al. 2009). The deeply diverged MYB12-like sequences are highlighted in dark yellow; *IcMYB12-like* corresponds to *Iochroma*.IC05g034110. Bolded branches have >95% bootstrap support; values between 50 and 95% bootstrap support are given. The outgroups used to root the tree (SG7 MYBs from *Arabidopsis*) have been pruned. b) Maximum likelihood phylogeny for Solanaceae *MYB12-like* sequences based on a complete CDS alignment. Bootstrap supports are shown as in (a). Tip values for *MYB12-like* expression (TPM) and floral flavonol content (in mg/g from Larter et al. 2019) for 6 species are colored by magnitude (see Supplementary Table 7 for raw data). These data are graphed in the inset figure with the dashed line showing the linear trend. *Iochroma cyaneum* is not included as data from previous transcriptomic analyses (Gates et al. 2018) are not directly comparable with the *de novo* transcriptomes from the present study. Full names and sources for all sequences used in these analyses are given in Supplementary Table 6. Branch lengths in both trees are in substitutions per site.

We used BLAST searches to retrieve similar sequences to *IcMYB12-like* (Supplementary Table 6). The top hits from *Arabidopsis* and Solanaceae genomes corresponded to members of subgroup 7 of R2R3 MYB transcription factors (Stracke et al. 2001). This subgroup controls flavonol production in *Arabidopsis* (Stracke et al. 2007) by regulating upstream steps such as CHS, CHI, and FLS, as well as the glycosyltransferases that stabilize these products. Maximum-likelihood analysis revealed that *IcMYB12-like* and highly similar sequences from pepper and potato are closely related to subgroup 7 but fall in a separate subclade, with strong support (Fig. 4a). Subgroup 7 MYBs have been well characterized in Solanaceae (e.g. Ballester et al. 2009; Song et al. 2020) and appear functionally similar to their orthologs in *Arabidopsis*. *Iochroma* possesses an SG7 MYB that is closely related to these well-characterized MYB12 genes (IC05g030210, Fig. 4a) in addition to the divergent sequence (IC05g034110), which we refer to as a *MYB12-like* gene following the naming of *CaMYB12-like* in *Capsicum* (CA05g18430 in Fig. 4a).

Using additional BLAST searches beyond nightshade crops, we identified an additional member of the *MYB12-like* clade in the wolfberry genus *Lycium*, which we used to root the phylogeny including the additional Iochrominae sequences (Fig. 4b). The resulting topology is similar to the species tree (Deanna et al. 2019) although many of the branches in Iochrominae are unsupported given that the sequences present few differences (Supplementary Fig. 8). Examining the expression of *MYB12-like* in these taxa in relation to their floral flavonol production (Larter et al. 2019; Supplementary Table 7), we observed that species with higher *MYB12-like* expression also produce higher amounts of flavonols (Fig. 4b), which are mainly quercetin glycosides (Berardi et al. 2016). This pattern aligns well with the proposed function of *MYB12-like* in activating *F3′H*, which in turn produces DHQ, the precursor of quercetin (Fig. 1).

## Discussion

This study aimed to identify the gene underlying the so-called *T-*locus, which acts as a transcriptional regulator of *F3′H* to determine flower color in the nightshade genus *Iochroma*. By carrying out RNASeq of floral bud tissue from multiple backcross individuals with *T-*locus genotypes assigned based on flower color (Fig. 1b), we pinpointed an R2R3 MYB transcription factor as the strongest candidate for the *T-*locus. First, our SNP-association studies narrowed the candidate region to 10Mb near the end of chromosome 5 (Fig. 2 and Supplementary Fig. 4). This region of the genome contains 468 gene models, including 28 annotated as transcription factors. Among these, only one of these corresponds to a class of genes, SG7 MYBs, known to be involved in regulating flavonoid biosynthesis. This *MYB12-like* gene shows tightly correlated expression with *F3′H* across the backcross (*r* = 0.91, *P* = 1.58 ×10^−05^, Fig. 3a and b). Indeed, these 2 genes emerge as part of a compact module in transcriptome-wide co-expression analyses, with *F3′H* having a stronger connection with *MYB12-like* than any other gene in the floral transcriptome with the exception of *FLS* (Fig. 3b and Supplementary Fig. 6). Given that the effect of the *T*-locus could be due to coding sequences changes only, this set of analyses cannot conclusively eliminate other candidate transcription factors in the associated region of the genome. Nevertheless, our phylogenetic analyses identify *IcMYB12-like* as an ortholog of chillipepper *CaMYB12-like*, a recently characterized flavonoid regulator, which like its *Iochroma* ortholog, acts as a positive regulator of *F3′H* (Wu et al. 2023). Together, these lines of evidence argue that the *T-*locus corresponds to the *MYB12-like* gene in *Iochroma*, which drives the origin of red flowers by altering floral flavonoid composition. Below, we discuss how these findings contribute to our broader understanding of flower color evolution.

### Genetic basis of changes in floral hue

While genetic studies of flower color have long implicated subgroup 6 R2R3 MYBs as the major determinants of floral pigment intensity (e.g. Quattrocchio et al. 1999; Schwinn et al. 2006; Streisfeld et al. 2013; reviewed in Marin-Recinos and Pucker [2024]), work on the genetic basis of changes in floral hue has implicated a wide variety of molecular mechanisms (Quattrocchio et al. 2006; Wessinger and Rausher 2012; Berardi et al. 2021). Differences in the type of anthocyanins produced, which in turn influence the type of flower color, can arise from shifts in gene regulation (either in *cis*- or *trans*-) as well as changes in the function of pathway enzymes (Smith and Rausher 2011; Hopkins and Rausher 2011; Smith et al. 2013; Wessinger and Rausher 2015; Wheeler et al. 2023). Nevertheless, the identity of transcription factors that influence the type of anthocyanin produced (as opposed to the overall amount) through changes in *F3′H* and/or *F3′5′H* expression has remained nebulous, even in well-studied model systems.

Because of the shared precursors within the flavonoid pathway, subgroup 7 (SG7) transcriptional regulators of flavonol production can directly influence anthocyanin production, and, as shown in the present study, the type of anthocyanin produced as well. The deeply conserved structure of the pathway presents multiple branching points where a single precursor can be converted in different products depending on the enzymes present and their properties (Winkel-Shirley 2001; Tohge et al. 2013). The colorful anthocyanins share dihydroflavonol precursors (DHK, DHQ, DHM) with flavonols, creating the potential for competition between DFR and FLS for these substrates (Fig. 1a). Thus, the upregulation of SG7 MYBs and, in turn, their targets (e.g. *CHS*, *CHI*, *F3H*, *FLS*) generally reduces anthocyanin production in favor of flavonols to produce paler flowers (Holton et al. 1993; Luo et al. 2016; Yuan et al. 2016; Wheeler et al. 2023). The precise effect of altering the expression of SG7 MYBs on flower color will, however, depend on their target genes and the substrate preferences of multifunctional pathway enzymes (e.g. DFR, FLS). The degree of competition for shared precursors may be mitigated by reduced spatiotemporal overlap in expression and/or functional specialization on different precursors (e.g. FLS on DHK and DFR on DHQ; Choudhary and Pucker 2024).

In the case of *Iochroma*, the ability of the SG7 *MYB12-like* gene to alter flower color is likely due to the combination of a narrowing of target genes and strong substrate preferences among downstream enzymes. While the pepper *CaMYB12-like* gene activates a broad suite of pathway genes (*CHS, CHI, F3H, F3′H, FLS,* UGT, Wu et al. 2023), the *Iochroma* ortholog only shows strong co-expression with *F3′H* and *FLS* (plus weaker co-expression with *CHS* and *UGT*), pointing to a reduced suite of targets, a functional shift that could be further explored with promoter-binding assays or transient transformation (e.g. Mehrtens et al. 2005; Yang et al. 2023). The broad upstream action by *CaMYB12-like* is similar to that of the other well-known SG7 MYBs in Solanaceae (*SlMYB12* in tomato, Ballester et al. 2009; Fernandez-Moreno et al. 2016; *NtMYB12* in tobacco, Song et al. 2020), suggesting that coordinated regulation of the first steps of the flavonoid pathway represents the ancestral state and that the functional shift toward specificity has occurred along the *MYB12-like* lineage leading to *Iochroma*. Accordingly, the loss of *MYB12-like* expression in *I. gesnerioides* flowers is not associated with a complete disruption in floral flavonoid pigment production (Larter et al. 2019; Berardi et al. 2021), but a targeted reduction in DHQ through lower *F3′H* expression. The resulting accumulation of DHK is not converted to kaempferol, likely because of coordinated loss of *FLS* expression. Instead, this DHK precursor is converted to red pelargonidin pigments by the DFR enzyme, which in *I. gesnerioides*, is specialized for DHK (Smith et al. 2013). Smith et al. (2013) hypothesized that, during the evolutionary transition from blue to red flowers, the *trans*-regulatory loss of *F3′H* expression occurred first, allowing the flux to shift toward red pigmentation. Under this scenario, the selection would be expected to favor increased activity of DFR on DHK to allow efficient conversion to red pelargonidins.

This *MYB12-like*-mediated biochemical trade of blue anthocyanins plus flavonols for red anthocyanins alone may have also carried ecological consequences for relationships with pollinators. In addition to acting as co-pigments, flavonols increase floral UV-absorbance, which is attractive to moth pollinators (Sheehan et al. 2016) and can also enhance fly and bee visitation if associated with floral patterning (Koski and Ashman 2014). Indeed, insects comprise only 10% of pollinator visits to *Iochroma gesnerioides* compared hummingbirds, which account for 90% of visits (Smith et al. 2008). This lack of UV-absorbing flavonols is isolated to *I. gesnerioides* flowers as the leaves produce comparable amounts of flavonols (specifically quercetin) as the blue-flowered *I. cyaneum* (Berardi et al. 2016) and the expression of *F3′H* is actually higher in *I. gesnerioides* leaves than in those of *I. cyanuem* (Smith and Rausher 2011). The targeted effects of *MYB12-like* on floral flavonols may have thus created an accessible evolutionary pathway to red flowers, given that a loss of quercetin across the entire plant would carry significant negative pleiotropic effects (Ryan et al. 2001; Singh et al. 2021).

### MYB transcription factors in the evolution of species differences

Closely related species of flowering plants are often distinguished by subtle differences in their reproductive organs, e.g. in the color, shape, scent, or pubescence of flowers or fruits. MYB transcription factors control many of these aspects of morphological development and epidermal cell fate (Ramsay and Glover 2005; Hileman 2014), which may help to explain their prevalence in underlying fixed differences between species (e.g. Preston et al. 2011; Gates et al. 2018; Castillejo et al. 2020; Yarahmadov et al. 2020). In fact, MYB transcription factors may act as speciation genes when the phenotypic differences resulting from changes in their function or expression leads to reproductive isolation (Streisfeld et al. 2013; Sheehan et al. 2016; Lüthi et al. 2022). Through its simultaneous effects on visible anthocyanins and UV-absorbing pigments, changes in floral *MYB12-like* expression could have played a role in species divergence, although the split between the red-flowered clade containing *I. gesnerioides* and its blue-flowered relatives likely occurred 5 to 10 million years ago (Huang et al. 2023), and the 2 lineages no longer occur in hybrid zones. The *I. arborescens* complex (the “A” clade sensu Smith and Baum 2006) presents a stronger opportunity for dissecting the role of *MYB12-like* in floral isolation as red-flowered, low-flavonol primarily-hummingbird-pollinated species (e.g. *I. edule*) co-occur and hybridize with flavonol-rich insect-pollinated species (e.g. *I. arborescens*) (Smith et al. 2008; Fig. 4b).

*Cis*-regulatory mutations involving MYBs appear to be a major target for evolutionary transitions, and our results suggest that regulatory changes, as opposed to functional variation, drive the effects of *MYB12-like* on flower color in *Iochroma*. First, the *MYB12-like* sequence from *I. gesnerioides* shows 6 fixed amino acid differences from its closest blue-flowered relative (*I. calycinum*); however, all but one of these variants (a threonine indel close to the 3′ end, Supplementary Fig. 8) are segregating across *Iochroma* species with both low and high flavonol accumulation (Fig. 4b). Moreover, *MYB12-like* expression levels are strongly predictive of pathway activity. Within the backcross, the red *I. gesneroides* parent allele of *MYB12-like* is expressed at extremely low levels, which in homozygous state translates to a near absence of *F3′H* expression (Fig. 3a). This relationship extends above the species level, where lower levels of *MYB12-like* expression appear to be associated with lower levels of quercetin flavonols (Fig. 4b). Beyond *Iochroma*, *cis-*regulatory mutations at MYB transcription factors frequently contribute to within and among species differences in flower color (Streisfeld et al. 2013; Martins et al. 2017; Fattorini and Ó’Maoiléidigh 2022), a pattern that has been attributed to their comparatively limited pleiotropic effects (Sobel and Streisfeld 2013). Nevertheless, the precise changes in the MYB promoters are unknown in these natural systems. Identifying the causal mutation(s) will require fine dissection of the promoter region along with *in vivo* or *in vitro* assays of various constructs (e.g. Espley et al. 2009; Jia et al. 2021). Although transformation remains challenging outside of model systems, pinpointing these causal variants is important for ultimately understanding how and why MYBs and the modules they control can be deployed in new developmental contexts.

## Conclusions

As a powerful group of antioxidants, flavonols have long been the focus of efforts in plant breeding, resulting in a detailed understanding of the subgroup 7 MYBs that largely control their expression across flowering plants. Within the nightshades, the best known of these MYBs are the orthologs of MYB12, which contribute to stress tolerance in tobacco (Song et al. 2020) and the color of the fruit peel in tomato (Ballester et al. 2009) via their effects on flavonoid production. This gene family is also expressed in Solanaceae flowers (Zheng et al. 2021), activating multiple branches of the pathway to provide both flavonol co-pigments and the substrates for anthocyanin biosynthesis. Our work reveals that, in addition to this canonical “MYB12' group of SG7 MYBs, *Iochroma* flowers also express a more divergent “MYB12-like” lineage that has evolved narrow specificity for *FLS* and *F3′H*. This specialization, together with flower-specific expression, allows *IcMYB12-like* to act as the switch between blue and red flowers. Piecing together the origin of this gene's role in floral flavonoid production will require targeted comparative genomic, transcriptomic, and metabolomic studies to localize the timing of this functional shift within the nightshades, as well as additional experimental work to identify the upstream regulators of flavonoid MYBs and their binding motifs within *cis*-regulatory regions (e.g. Chopy et al. 2024). Overall, our study underscores the importance of studying flower color evolution in the context of the entire flavonoid pathway as its conserved branching architecture shapes trade-offs between the visible pigments and “colorless” compounds.

## Supplementary Material

jkaf230_Supplementary_Data

## Data Availability

RNA-seq data from the backcross individuals have been uploaded to the SRA under Bioproject PRJNA1092111. Data for the other 6 species have been uploaded to SRA Bioproject PRJNA1102413. Code and additional data used in the analyses are available at DOI 10.17605/OSF.IO/J5M8F. Code for *de novo* transcriptome assembly are available through the repository associated with Wheeler et al. (2022), https://osf.io/b7gcp/. Supplemental material available at G3 online.
