# Supplementary material for: MYB regulator of “colorless” flavonols underlies the evolution of red flowers in Iochroma (Solanaceae)
Source: G3 (Bethesda). 2025 Sep 30;15(12):jkaf230. doi: 10.1093/g3journal/jkaf230 (PMC12693573; doi:10.1093/g3journal/jkaf230)
Supplement: jkaf230_Supplementary_Data [file jkaf230_supplementary_data.zip › Supplementary_Figures_G3-2025-406150.docx]

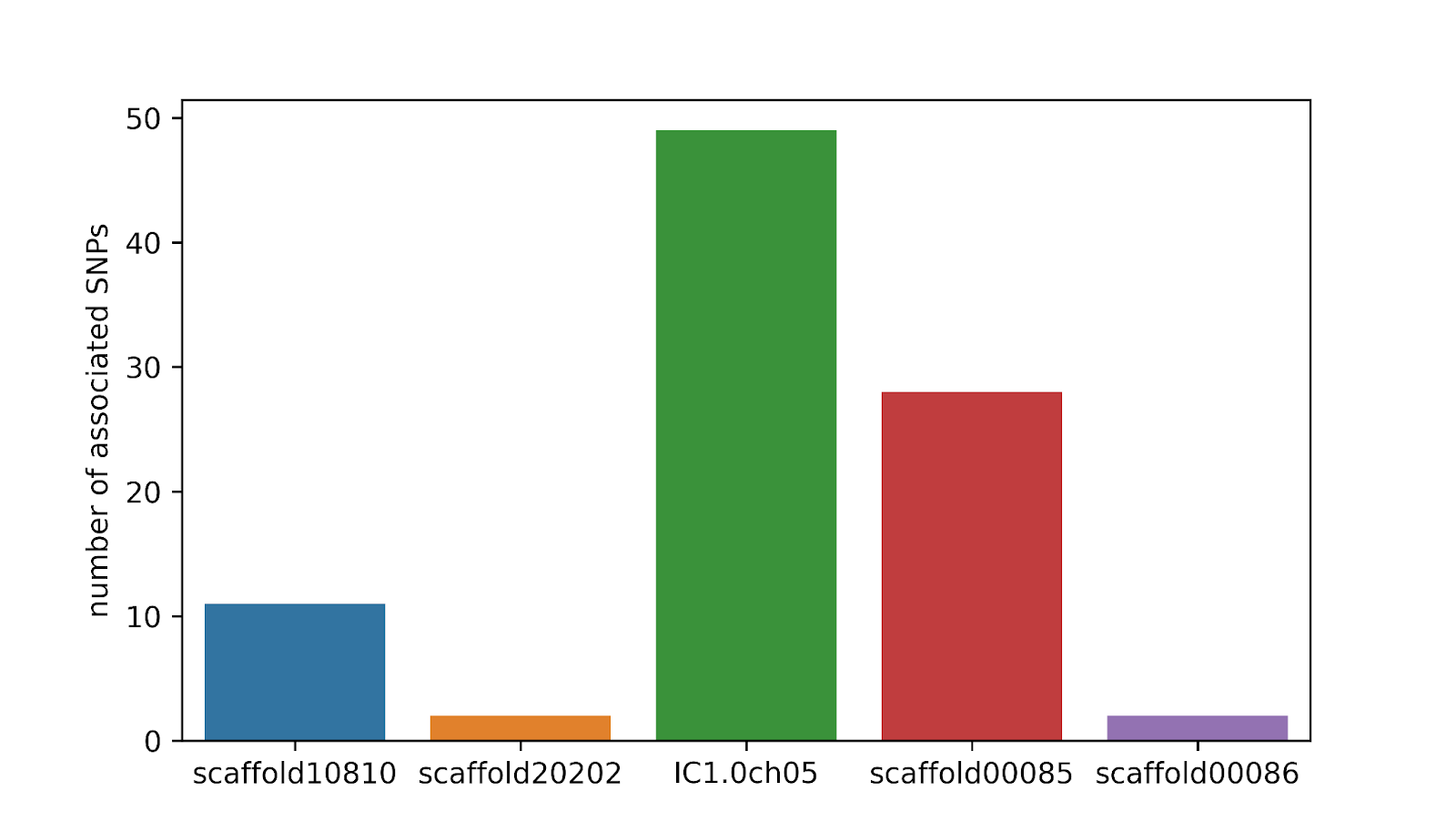


**Supplementary Fig. 1.** The scaffolds containing SNPs perfectly associated with the two phenotypic pools (Fig. 1). The majority of the hits fall on *Iochroma cyaneum* chromosome 5 (IC1.0ch05). The largest scaffold is 00085 (619,457bp), followed by 10810 (4263 bp), 20202 (15763 bp), and 00086 (159389 bp). All of these scaffolds have top BLAST hits to IC1.0ch05, suggesting they belong in that chromosome and were simply unincorporated in the assembly process.


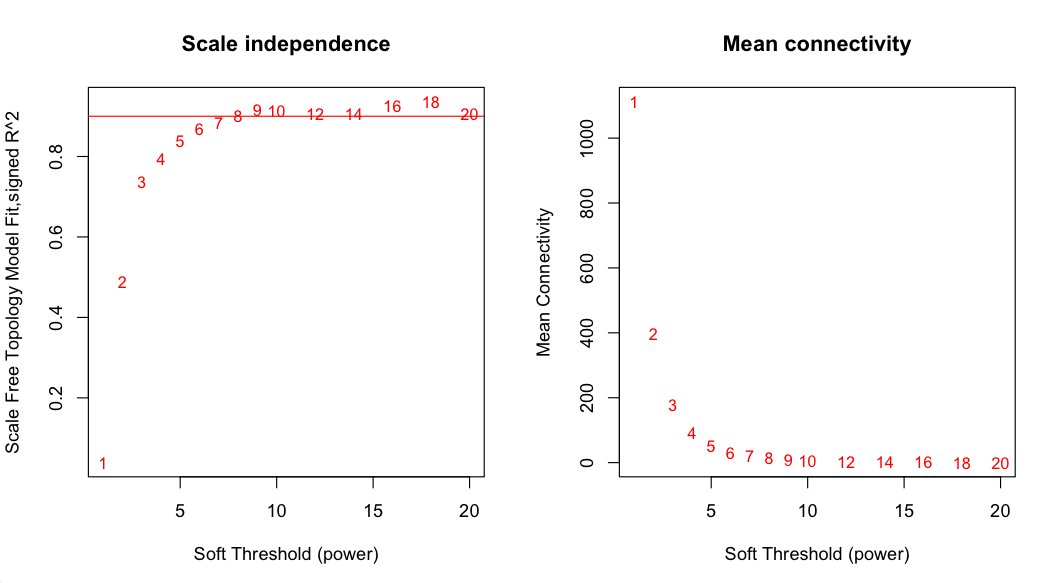


**Supplementary Fig. 2.** Scale independence and mean connectivity for soft threshold selection for WGCNA analysis. Left graph shows the fit to the scale-free topology model across soft threshold values (red horizontal line corresponds to R^2^ value of 0.9). Right graph shows mean values for connectivity associated with soft threshold values.


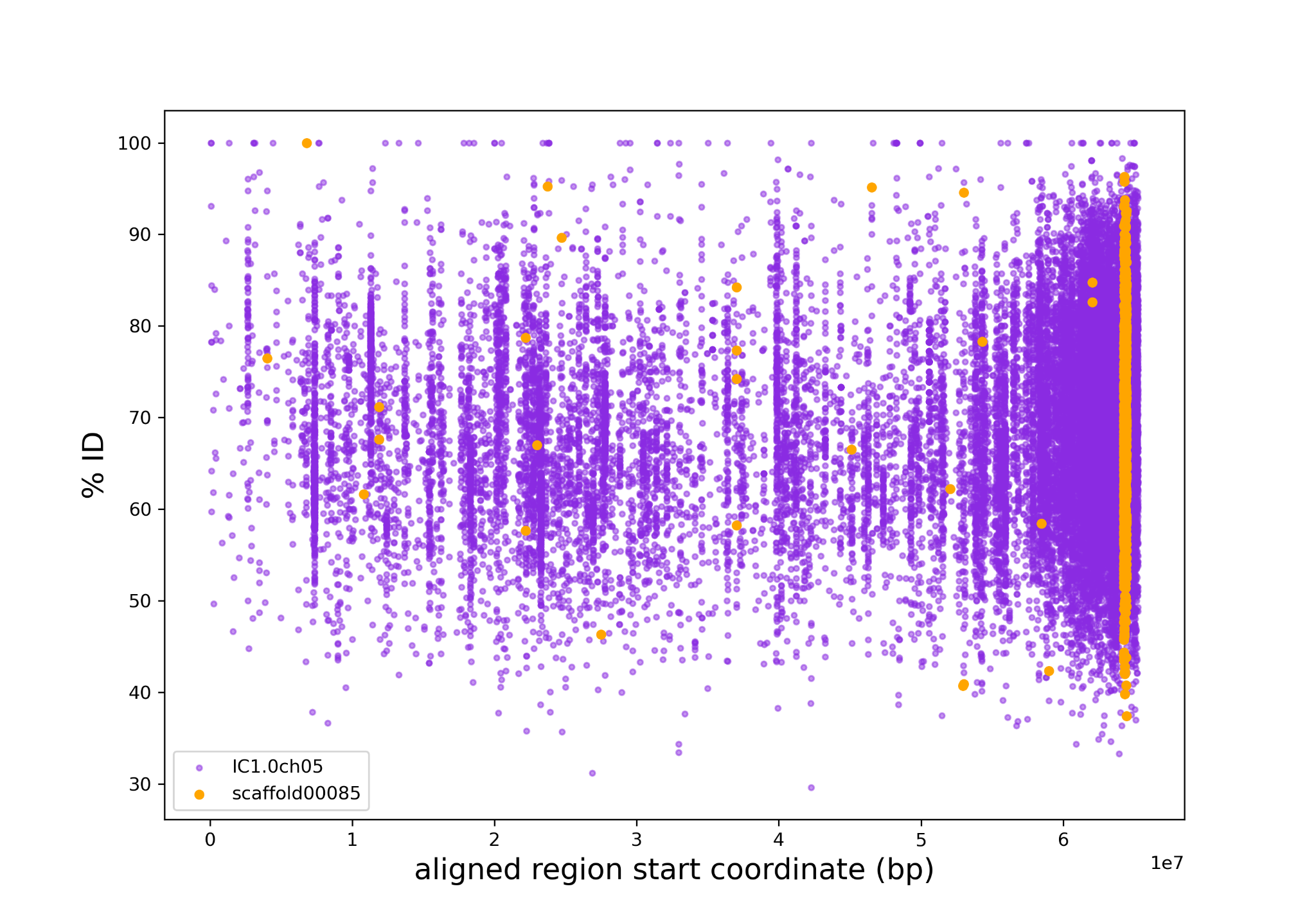


**Supplementary Fig. 3.** *Iochroma* chromosome 5 and scaffold00085 mapped to tomato chromosome 5. Purple points show the mapping across genomic windows from *I. cyaneum* chromosome 5 to tomato chromosome 5 (SL4.0). Scaffold00085 from *I. cyaneum* contains 28 SNPs associated with the color phenotype, and it maps to the 3’ end of tomato chromosome 5 (orange points). Its synteny with tomato suggests that scaffold00085 also belongs to *I. cyaneum* chromosome 5 and sits in the region associated with flower color.


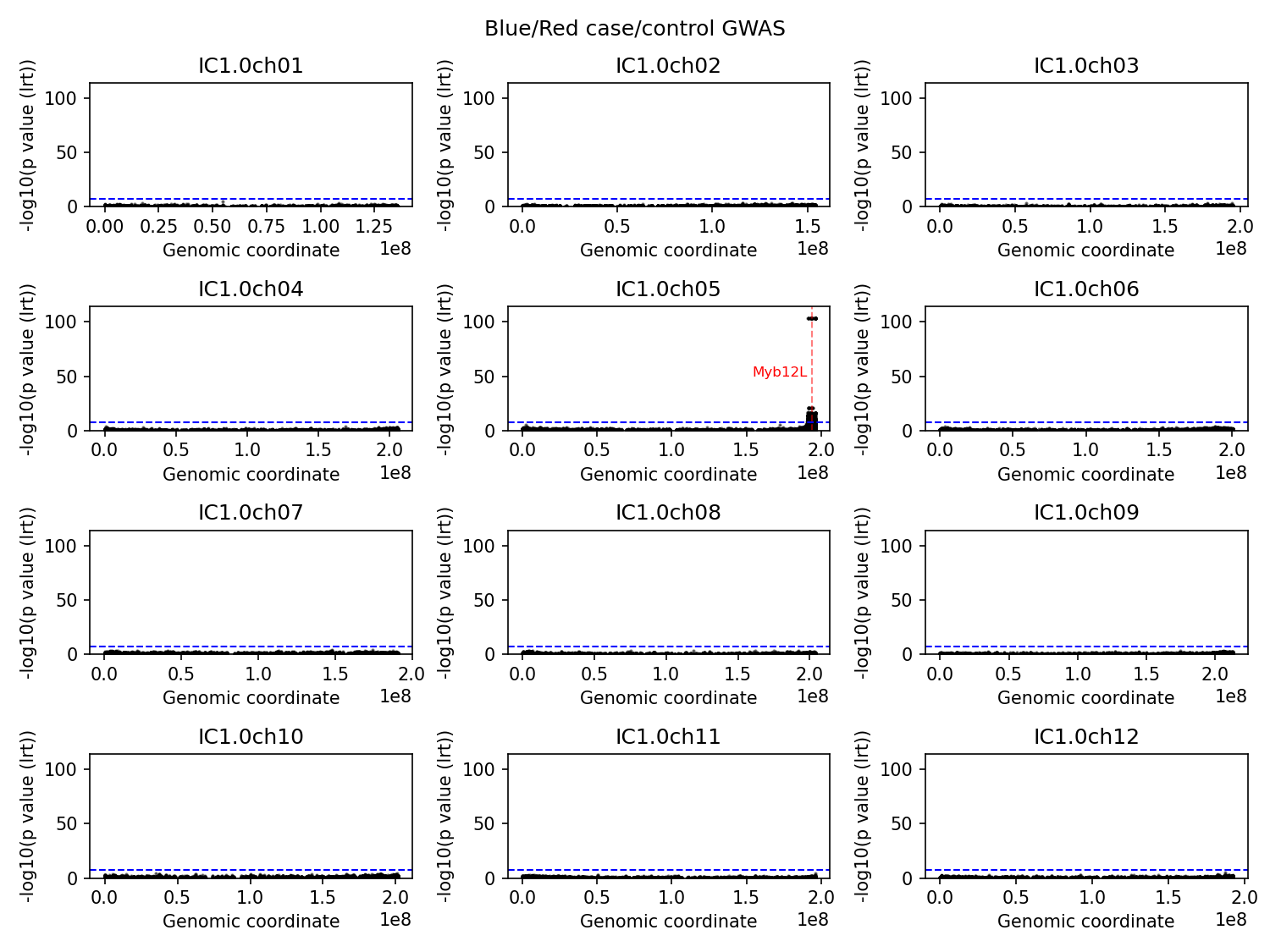


**Supplementary Fig. 4.** Manhattan plots for associations with flower color across all *I. cyaneum* chromosomes. The blue dashed lines indicate genome-wide significance at P<5x10^-8^. Genomic coordinates are indicated along the x-axes in Mb.


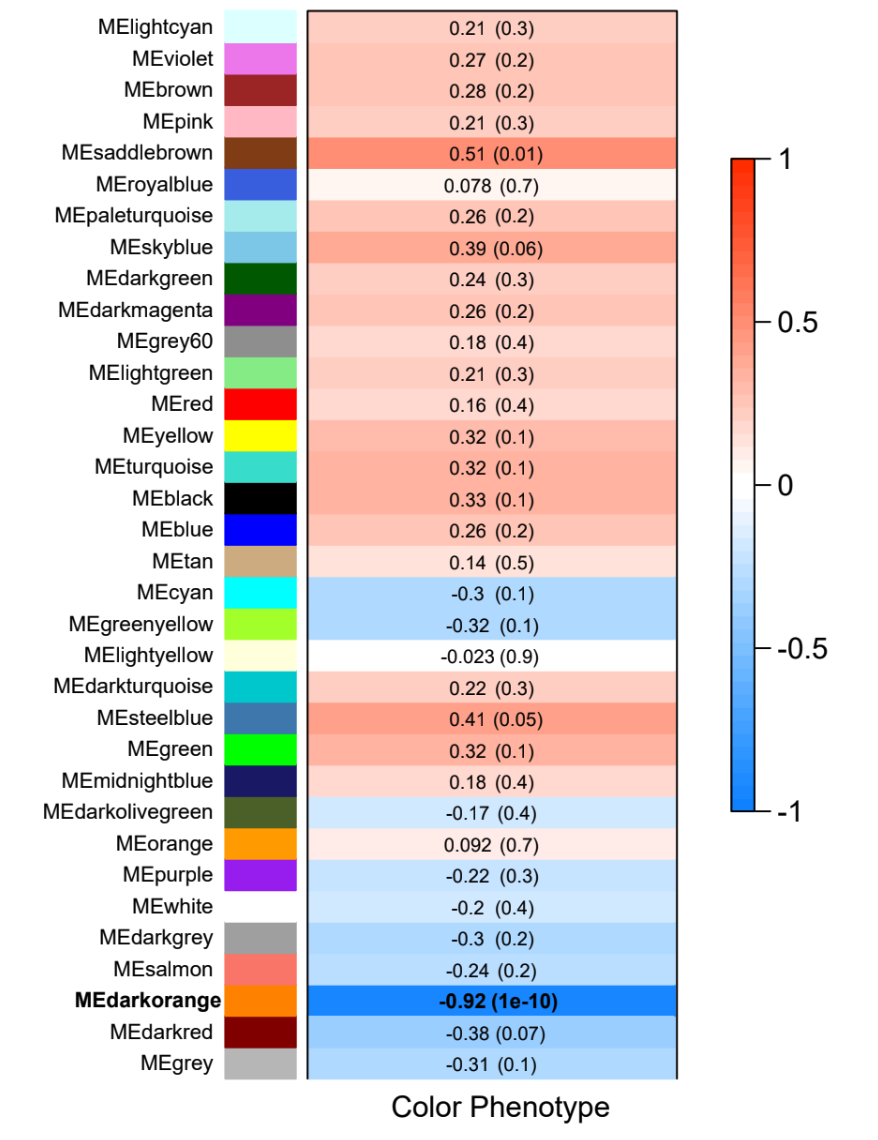


**Supplementary Fig. 5.** Module-trait relationships from the WGCNA analysis. The plot shows the correlations of gene modules with the color phenotype (purple vs. pink/red, Fig. 1). The numbers in each block represent the correlation coefficient and P-value (parentheses). Cells are colored by the value of the correlation coefficient. Only the bolded value (‘dark orange’ module) is significant after Bonferroni correction.


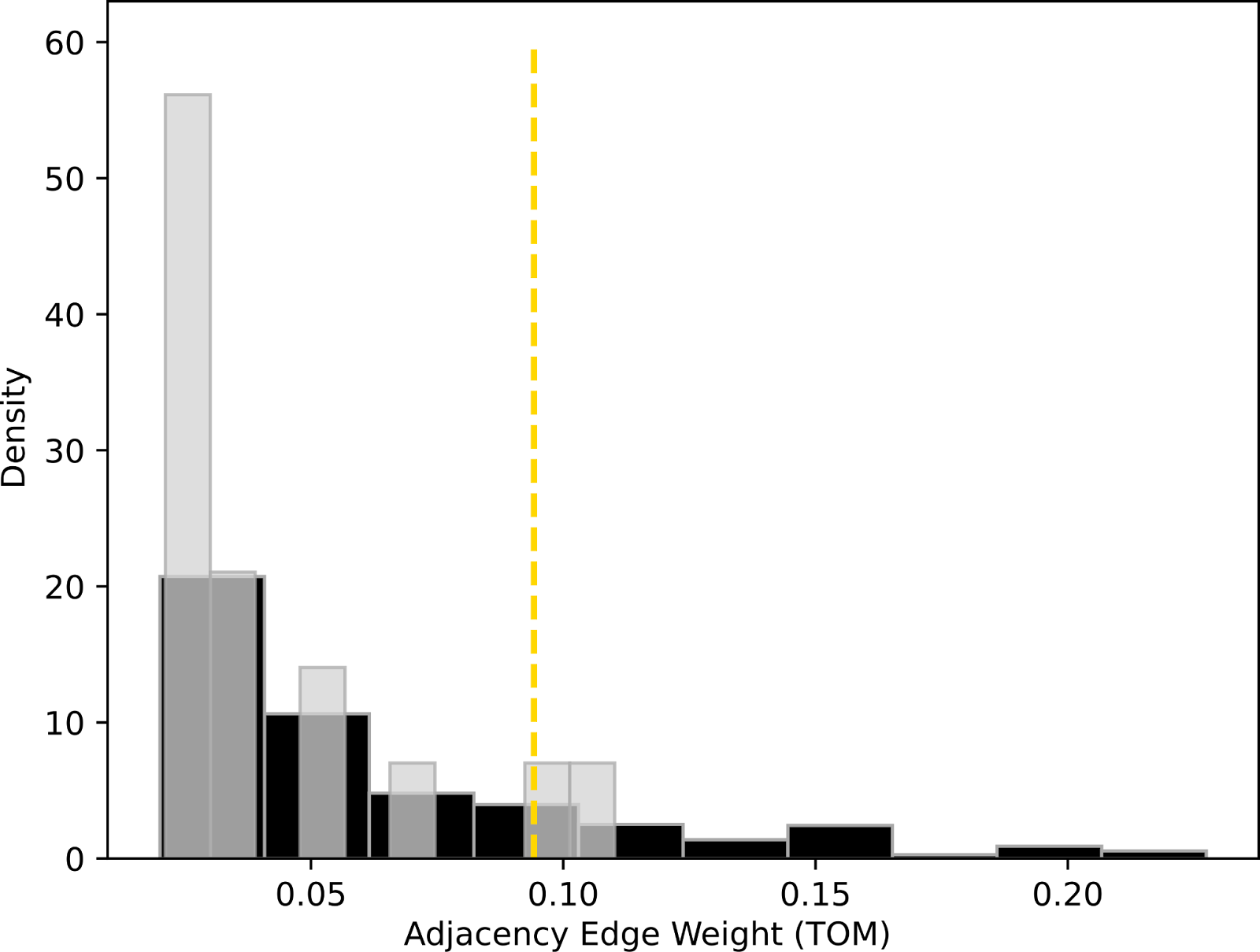


TOM values

**Supplementary Fig. 6**. **Distribution of connectivity (TOM) values between DE transcripts**. The Topological Overlap Matrix (TOM) is calculated from the adjacency matrix by dividing the sum of adjacency weights of a gene's neighbors by the total number of neighbors. The black bars in histogram show the overall distribution of TOM values in the module associated with the phenotype (Table S4, Fig. S5) from the WGCNA analysis. Gray bars show coefficients for just the edges involving *F3'h.* The orange dashed line highlights the connection between *F3’h* and *IcMYB12-like*. The y-axis shows the number of edges in each TOM value bin.


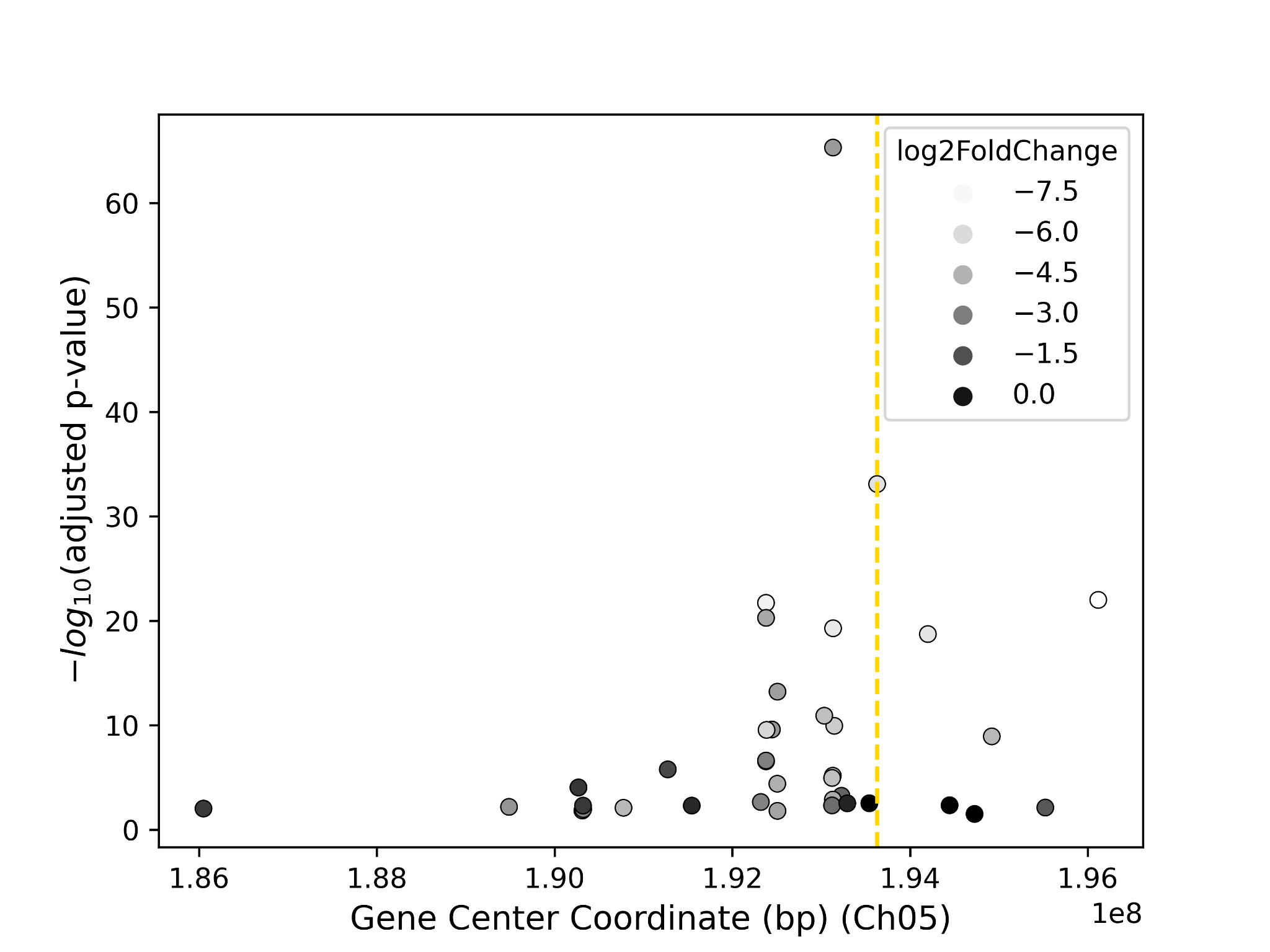


**Supplementary Fig. 7. Elevated DE near *MYB12-like* locus**. Significantly differentially expressed transcripts from the DESeq analysis were mapped to the genomic region containing *MYB12-like* (denoted with the gold dashed line). Each point represents a transcript, shaded by the degree of differential expression (log_2_-fold-change). The y-axis shows the -log_10_ of the adjusted p-value.


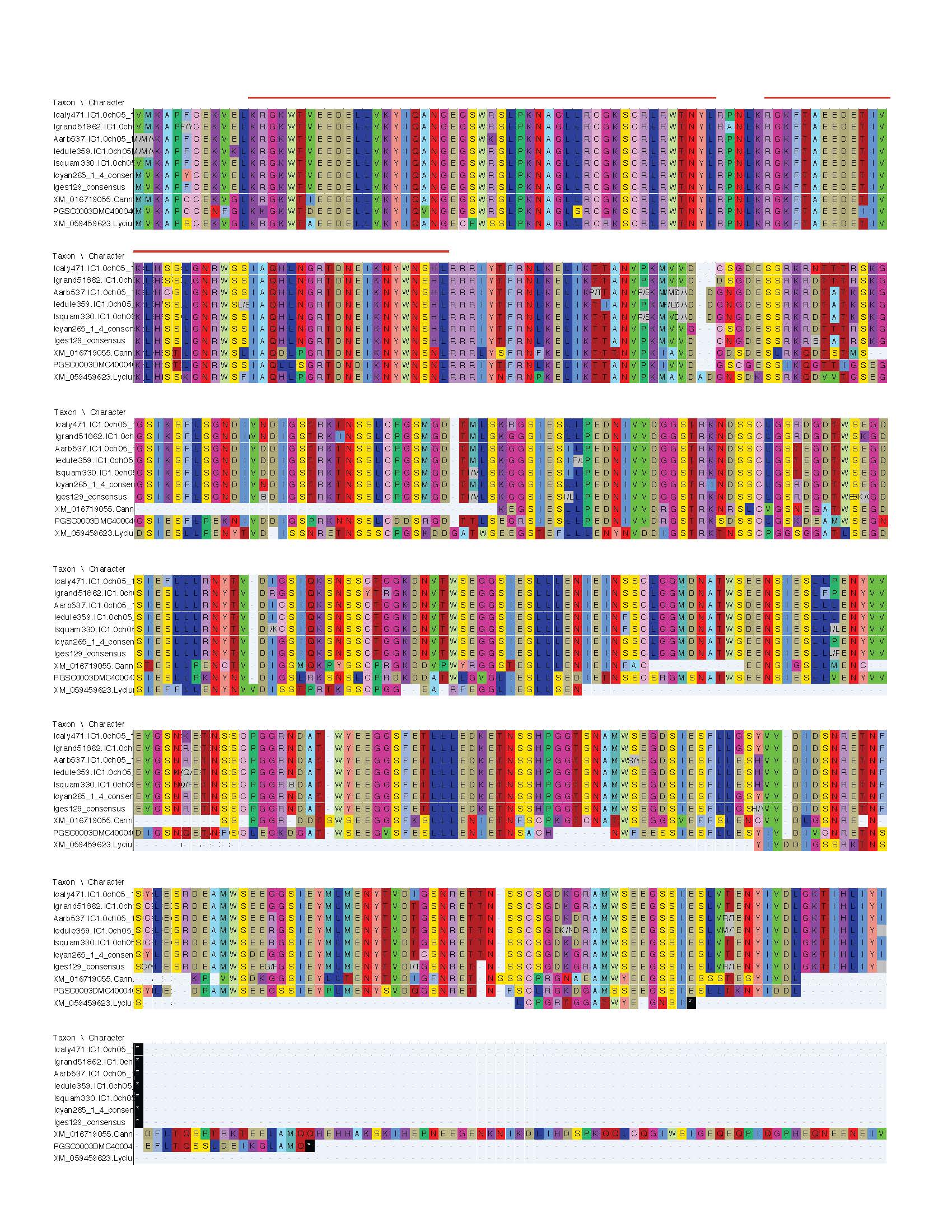


**Supplementary Fig. 8. Amino acid alignment for MYB12-like sequences from Iochrominae and other Solanaceae**. The R2 and R3 domains are denoted with red bars. Full names and sources for all sequences used in this analysis are given in Supplementary Table 7.
