# Supplementary material for: MYB regulator of “colorless” flavonols underlies the evolution of red flowers in Iochroma (Solanaceae)
Source: G3 (Bethesda). 2025 Sep 30;15(12):jkaf230. doi: 10.1093/g3journal/jkaf230 (PMC12693573; doi:10.1093/g3journal/jkaf230)
Supplement: jkaf230_Supplementary_Data [file jkaf230_supplementary_data.zip › Supplementary_Table_Legends_G3-2025-406150.docx]

**Supplementary Table Legends**

**Supplementary Table 1.** Gene models in associated region. List of 469 gene models and their coordinates in the *Iochroma* genome.

**Supplementary Table 2.** Functional annotations of gene models. Predicted function for each gene in the associated region based on BLAST.

**Supplementary Table 3.** DESeq Results. Differentially expressed genes between the two phenotypic pools (Fig. 1). Log-fold change in expression is given for all transcripts. The adjusted p-value corresponds to a false discovery rate of 5%.

**Supplementary Table 4.** Pairwise correlations between the expression of *F3'H* and other genes. Raw and Bonferroni-corrected p-values are given for each comparison.

**Supplementary Table 5.** Phenotype-associated model from WGCNA analysis. Genes present in module associated with the phenotype. Connectivity values are given for each pair of co-expressed genes.

**Supplementary Table 6.** Metadata for sequences used in phylogenetic analyses, including accession numbers, annotations and links to sources.

**Supplementary Table 7.** *MYB12-like* expression and flavonol production. Values for *MYB12-like* expression (TPM) and floral flavonol production (mg/g) across sampled *Iochroma* species.
